# Supplementary material for: Features and signals in precocious citation impact: A meta-research study
Source: PLoS One. 2025 Aug 6;20(8):e0328531. doi: 10.1371/journal.pone.0328531 (PMC12327621; doi:10.1371/journal.pone.0328531)
Supplement: Attachment — Submitted filename: responsesplosoneprecocious.docx [file pone.0328531.s002.docx]

May 29, 2025

Miquel Vall-llosera Camps

Senior Staff Editor

PLOS ONE

PONE-D-24-56818

Features and signals in precocious citation impact: a meta-research study

Dear Editor

I was pleased to hear that PLoS One is interested in a revised version of this manuscript. I am grateful for the insightful comments of the reviewers. I have addressed all of them in the current revision. In more detail.

Journal Requirements:

When submitting your revision, we need you to address these additional requirements.

1. Please ensure that your manuscript meets PLOS ONE's style requirements, including those for file naming. The PLOS ONE style templates can be found at

https://journals.plos.org/plosone/s/file?id=wjVg/PLOSOne_formatting_sample_main_body.pdf and

https://journals.plos.org/plosone/s/file?id=ba62/PLOSOne_formatting_sample_title_authors_affiliations.pdf

Reply: done

2. We note that the grant information you provided in the ‘Funding Information’ and ‘Financial Disclosure’ sections do not match.

When you resubmit, please ensure that you provide the correct grant numbers for the awards you received for your study in the ‘Funding Information’ section.

Reply: these have been aligned. Some funds have no grant number.

3. Thank you for stating the following financial disclosure:

“The work of John Ioannidis is supported by an unrestricted gift from Sue and Bob O’Donnell to Stanford University.”

Please state what role the funders took in the study. If the funders had no role, please state: "The funders had no role in study design, data collection and analysis, decision to publish, or preparation of the manuscript." If this statement is not correct you must amend it as needed.

Please include this amended Role of Funder statement in your cover letter; we will change the online submission form on your behalf.

Reply: The funders had no role in study design, data collection and analysis, decision to publish, or preparation of the manuscript.

4. Please note that funding information should not appear in the Acknowledgments section or other areas of your manuscript. We will only publish funding information present in the Funding Statement section of the online submission form. Please remove any funding-related text from the manuscript.

Reply: Removed.

5. Thank you for stating the following in the Competing Interests section:

“METRICS has been funded by grants from the Laura and John Arnold Foundation (Arnold Ventures).”

Please confirm that this does not alter your adherence to all PLOS ONE policies on sharing data and materials, by including the following statement: "This does not alter our adherence to PLOS ONE policies on sharing data and materials.” (as detailed online in our guide for authors http://journals.plos.org/plosone/s/competing-interests). If there are restrictions on sharing of data and/or materials, please state these. Please note that we cannot proceed with consideration of your article until this information has been declared.

Please include your updated Competing Interests statement in your cover letter; we will change the online submission form on your behalf.

Reply: This does not alter our adherence to PLOS ONE policies on sharing data and materials.

6. Please note that your Data Availability Statement is currently missing the repository name and direct link to access each database. If your manuscript is accepted for publication, you will be asked to provide these details on a very short timeline. We therefore suggest that you provide this information now, though we will not hold up the peer review process if you are unable.

Reply: As I state “All the raw data are available in pre-existing publicly available databases (see links in the text) and in the manuscript.”.

Additional Editor Comments:

I would like to sincerely apologise for the delay you have incurred with your submission. It has been exceptionally difficult to secure reviewers to evaluate your study. We have now received two completed reviews; the comments are available below. The reviewers have raised significant scientific concerns about the study that need to be addressed in a revision.

Please revise the manuscript to address all the reviewer's comments in a point-by-point response in order to ensure it is meeting the journal's publication criteria. Please note that the revised manuscript will need to undergo further review, we thus cannot at this point anticipate the outcome of the evaluation process.

Reply: Thank you for making an extra effort to secure reviewers. The comments are excellent and very helpful. Please see the point by point responses below.

Reviewers' comments:

Reviewer's Responses to Questions

Comments to the Author

1. Is the manuscript technically sound, and do the data support the conclusions?

The manuscript must describe a technically sound piece of scientific research with data that supports the conclusions. Experiments must have been conducted rigorously, with appropriate controls, replication, and sample sizes. The conclusions must be drawn appropriately based on the data presented.

Reviewer #1: Partly

Reviewer #2: Yes

________________________________________

2. Has the statistical analysis been performed appropriately and rigorously?

Reviewer #1: I Don't Know

Reviewer #2: Yes

________________________________________

3. Have the authors made all data underlying the findings in their manuscript fully available?

The PLOS Data policy requires authors to make all data underlying the findings described in their manuscript fully available without restriction, with rare exception (please refer to the Data Availability Statement in the manuscript PDF file). The data should be provided as part of the manuscript or its supporting information, or deposited to a public repository. For example, in addition to summary statistics, the data points behind means, medians and variance measures should be available. If there are restrictions on publicly sharing data—e.g. participant privacy or use of data from a third party—those must be specified.

Reviewer #1: Yes

Reviewer #2: Yes

________________________________________

4. Is the manuscript presented in an intelligible fashion and written in standard English?

PLOS ONE does not copyedit accepted manuscripts, so the language in submitted articles must be clear, correct, and unambiguous. Any typographical or grammatical errors should be corrected at revision, so please note any specific errors here.

Reviewer #1: No

Reviewer #2: Yes

________________________________________

5. Review Comments to the Author

Please use the space provided to explain your answers to the questions above. You may also include additional comments for the author, including concerns about dual publication, research ethics, or publication ethics. (Please upload your review as an attachment if it exceeds 20,000 characters)

Reviewer #1: The manuscript examines precocious citation impact at the individual level, i.e., researchers who attain very high citation rates early in the career. The manuscript is overall interesting but has some issues mainly in the methods section. Many of them has to do with presentation and a lack of information and detail. Hopefully my questions and comments can help the author to improve the manuscript.

Reply: Thank you for the very helpful comments. Please see responses and revisions delineated below.

Abstract

The readability of the abstract could be increased by imposing conciseness (less detail, more substance with a focus on conclusions), better structure, and including the aim of the study.

Reply: the abstract has been revised accordingly.

Method

The operationalisations of “precocious citation impact” as top-cited within 8 years and “ultra-precocious citation impact” as top-cited within 5 years seems a bit arbitrary. It is not clear to me why the concept of precocious citation impact is operationalised this way? What assumptions and definitions are these operationalisations based on? My recommendation is that the author elaborate on this issue in the manuscript. (page 4) The author mentions this issue in limitations (page 15) but this should receive attention and clarification and be discussed in the methods section.

Reply: I have clarified upfront that: “The use of 8- and 5-year cut-offs is arbitrary. These thresholds were pre-set in the analysis so as to capture individuals who achieve extreme acceleration of their citation impact in the very early stages of their career placing them at the far end of the distribution of all scientists in this aspect. If one assumes that extremes are populated by a mixture of individuals with truly exceptional ability and others with manipulative (or even fraudulent behavior), it is possible that the relative share of the latter group may be enriched when an ultra-extreme threshold is used. Employing two thresholds also allowed to examine comparatively the two resulting cohorts of authors.”

The inclusion criteria based on “top-citedness” is not clear to me. An author is top-cited if the author is either among the top-2% of a composite citation indicator (it is unclear to me which population this percentile ranking is based on; this should be clarified in the manuscript), or among the top- 100000 authors based on the same composite citation indicator based on Scopus data. It is unclear to me how these two criteria for inclusion are related to each other. It seems as if the first is a relative cut-off threshold and the second is an absolute cut-off threshold. This makes me uncertain about to which extent these two operationalisations of “top-citedness” are comparable, and the extent to which they might refer two different definitions of top-citedness. My recommendation is that the author clarify and justify this operationalisation in the manuscript. (page 4) Overall there seem to be different operationalisations of top-citedness other than those mentioned above in the manuscript, e.g., data for 2018 and 2017 where only scientists reaching the top- 100000 ranks across all science are included (page 24). As a reader it is difficult to get a good overview of this issue, and I would recommend the author to clarify this in the manuscript. Furthermore, the use of the composite citation indicator should be justified in the manuscript. At page 14 in the discussion the indicator is justified to some degree, this should be done fully in the method section and further elaborated on.

Reply: I have clarified this as follows: “The composite indicator has been widely used in previous work and the respective datasets have been accessed over 4 million times to-date. In principle, it tries to amalgamate information on citations, co-authorship patterns, and authorship placement in positions that usually suggest greater contribution (single, first, senior author positions). An author is included if they manage to be in the top-2% of the composite indicator among all authors who have the same primary scientific subfield and have published at least 5 full papers. In addition, some authors are included because they are among the top-100,000 in the composite indicator across all authors in all science (roughly top-1%), even though they may not have made it to the top-2% among those who share the same primary scientific subfield.”

The inclusion criteria for being considered for ranking is “Scientists with (more or equal to) 5 papers are considered for ranking”, seems arbitrary. Why is this criterion imposed and how does it impact the ranking? What does “papers” mean here? Is it only based on peer reviewed journal articles? My recommendation is that the author justify this criterion in the manuscript. (page 4)

Reply: These criteria were set long ago on creating the composite indicator and have been extensively explained in previous work. I have nevertheless added: “the cut-off of at least 5 papers has been used since the original conception of the composite indicator. It aims to exclude authors with very limited presence in the literature. Articles, reviews, and conference papers are the only types of publications that count as papers.”

The author write: “Total number of top-cited authors in each annually released database allowed adjusting for increasing author numbers over time” (page 5). It is not clear to me why this is done or how this is done. Can the author elaborate a bit on this?

Reply: this is inherent to the selection using the top-2%, so if there are more total authors, it is still only the top-2% being selected.

The author write: “First, it was assessed whether they may have additional Scopus author ID records where early publications before 2018 (i.e., outside the 5-year frame) were included” (page 5). It is not clear to me how many authors where excluded due to this issue. This should be clearly stated in the manuscript. (page 5)

Reply: It is already stated in the Results that 2 authors were artefacts because of such split publication records.

The author write: “The eligible ultra-precocious scientists were then contrasted against other precocious authors (those starting to publish in 2015-2017) and the group of all top-cited authors (regardless of year of starting to publish) using the database that includes citation data to end of 2023” (page 5). Who are the authors starting 2015-2017? I cannot see that these cohorts have been mentioned previously in the manuscript.

Reply: Other precocious authors are those who are precocious but not ultra-precocious.

The author write: “The following descriptive characteristics were obtained and summarized as counts for discrete variables and median for continuous ones” (page 6). I cannot find this descriptive statistics in the manuscript.

Reply: this is what the Results section present, they are all descriptive statistics.

The cut-off threshold of >60 papers seems arbitrary (page 7). My recommendation is to justify this threshold with a reference of arguments.

Reply: this is a previously used cut-off, so I clarify that “the >60 cut-off has been used as a threshold of extreme publishing behavior in previous work (24”.

The author write: “For each of these signal indicators, the proportion of authors with the indicator was compared between ultra-precocious citation impact authors and all top-cited scientists. Given the exploratory nature of analyses, a conservative p-value<0.005 was considered statistically significant” (page 7-8). It is not clear to me why the significance level is set at 0.005 here. My recommendation is to justify it with a reference of arguments. What is the purpose of using p-values? What is the sample (what is it’s size) and to which population do the author wish to generalize? My recommendation is to clarify these issues in the manuscript.

Reply: A reference has been added to the classic paper (ref. 27) suggesting using 0.005 instead of 0.05. The sample here is the authors with precocious behavior versus other top-cited. These are exploratory analyses (hence 0.005 further justified), no power calculation was performed and I would avoid claiming one, this would be misleading.

To summarize, overall the methods section lacks information, clarity and detail on the definitions and operationalisations of precocious citation impact and top-citedness. These issues should be presented in the manuscript and not just referenced. Further, as a reader it is difficult to follow all the references to different databases and get a clear view of what the actual dataset/datasets that is used in the study and what they consist of. My recommendation is that the author makes an effort to increase the information, clarity and detail on how the dataset/datasets that is used in this study is constructed what it consists of in the methods section in a structured and clear fashion so that the transparency on these issues increases in the manuscript.

Reply: Thank you for the very helpful suggestions. Hopefully, with the additional referencing and clarifications, the information and clarity thereof should be improved now.

General questions:

Is precocious citation impact a phenomenon that is observable first and foremost at the individual level or is it relevant also at other levels, e.g, at document level or maybe research group level, etcetera.

Reply: I am not sure what “document level” would mean. For “research group level”, I have added: “the presented analyses focus on precocious citation impact as an individual author phenomenon. However, it is conceivable that the concept may be extended to research groups that extremely swiftly reach very high levels of impact. Precocious impact authors work within research teams. However, this is more difficult to study and calibrate, since there is no similar composite indicator ranking of impact for teams, as opposed to citation metrics and rankings for individuals. Nevertheless, some relevant precocious phenomena have been described for modest size teams and for entire universities. E.g. the advent of abruptly rising, massive trial publications with suspect characteristics from some center or author team (new reference 55) and the rapid growth of some institutions in number of affiliated highly cited scientists according to the Clarivate list of highly cited scientists (new reference 56).”

How does changes in the Scopus database, e.g., increased indexing over time, affect the longitudinal analyses?

Reply: There are more papers indexed over time indeed, but this does not necessitate precocious or extreme publishing behavior, it may have happened simply with more authors publishing.

This might not be possible, but, why do the author not examine the citing documents and their sources? From my point of view it would seem interesting to understand the citing side of this phenomenon.

Reply: This is interesting, but it would be a whole different project. I have added “In-depth assessment of single cases may also focus on assessing the citing documents, since this may reveal additional evidence for citation cartels or other questionable practices.”

Reviewer #2: The manuscript entitled “Features and signals in precocious citation impact: a meta-research study” addresses a highly relevant and timely topic: the emergence of patterns and signals of "precocious citation impact" and their potential associations with questionable scientific practices. The study is methodologically rigorous, employing multiple indicators to detect potential anomalies in citation trajectories. The writing is clear (although at times somewhat dense), and the overall approach is strong and systematic.

However, several aspects regarding the operationalization of key concepts, the interpretation of the findings, and the presentation of the results could be further strengthened to enhance the overall robustness, clarity, and impact of the study.

Reply: Thank you very much for the kind appreciation of this work. Please see responses and revisions listed below.

In my view, the following aspects could be further strengthened to enhance the manuscript:

- Definition of "Precocious Impact": While the selection of 8 and 5 years as thresholds for defining "precocious" and "ultra-precocious" citation impact is understandable, the manuscript would benefit from a more explicit justification of these specific cutoffs within the main text to contextualize the choice (especially, considering the diversity of fields considered in the study).

Reply: please see the reply to a similar comment made by Reviewer 1. I have added in the Methods upfront that “The use of 8- and 5-year cut-offs is arbitrary. These thresholds were pre-set in the analysis so as to capture individuals who achieve extreme acceleration of their citation impact in the very early stages of their career placing them at the far end of the distribution of all scientists in this aspect. If one assumes that extremes are populated by a mixture of individuals with truly exceptional ability and others with manipulative (or even fraudulent behavior), it is possible that the relative share of the latter group may be enriched when an ultra-extreme threshold is used. Employing two thresholds also allowed to examine comparatively the two resulting cohorts of authors.”

- Limitations of the signal indicators. One of the main risks associated with the use of these indicators is the potential for generalization and stigmatization. Given the notable overrepresentation of authors from less developed countries among those identified with precocious citation impact, the discussion should more explicitly address the risk of reinforcing existing structural biases or inequities in global science. Emphasizing the diversity of scientific excellence across different contexts would help provide a more balanced interpretation of the findings. Moreover, given the sensitive nature of using country affiliation as a signal, it would be important to clearly state that any future application of such indicators in research assessments must be accompanied by strong safeguards to prevent bias or discrimination based solely on an author's geographical origin.

Reply: This is a valid concern. I have added that “Future applications of the indicators of precocious impact should be accompanied by safeguards to prevent bias or discrimination based solely on an author’s geographical origin. Some truly excellent research and outstanding talent may be found in less developed countries.”

Now I will go briefly on more details.

Abstract

The abstract is generally clear and informative. However, I would recommend simplifying some of the longer sentences to improve readability. For example, the sentence "In-depth assessment of validated ultra-precocious scientists in 2023, showed significantly higher frequency..." is quite long and could be streamlined for clarity. Similarly, the phrase "The 17 ultra-precocious citation impact authors in the 2017-2020 top-cited lists who had retractions by October 2024..." could be simplified by removing details such as the timeline of retractions, which might be more appropriate for the main text rather than the abstract.

Reply: Thank you for these suggestions. The abstract has been edited accordingly.

Finally, the transition between "top-cited authors" and "Scopus author IDs" might cause minor confusion for readers unfamiliar with the methodology; clarifying or simplifying this distinction in the abstract would enhance accessibility (population should be clear).

Reply: indeed; this has now been clarified.

Methods

-Regarding the composite citation indicator, it would be helpful to clarify how co-authorship is weighted and how authorship placement (single, first, last) is specifically considered into the calculation. Additionally, when mentioning that "scientists with ≥5 full papers are considered for ranking," it would be important to specify whether all publication types (e.g., editorials, letters, commentaries) are included or if any types were excluded.

Reply: please see the added clarifications based on the similar suggestions of reviewer 1. I have also added that “Editorials, letter, and commentaries are not included.”

- Although the exploratory nature of the study is well justified, presenting a clearer flowchart or schematic overview of the analytical steps would greatly aid the reader in following the complex methodology.

Reply: The indicators are presented in numbering order and are also shown in the same order in all the relevant figures, so I doubt that repeating this information in yet another figure would help.

-A brief explanation of the c/h² orchestration index within the main text would be beneficial, particularly for readers who may not be familiar with this metric.

Reply: I have added “Authors with very low values of this metric have a disproportionately high h-index when contrasted to their total citations. This may mean that at least some of them may have self-cited or had other scientists or even fake papers cite their work in a way that it maximizes the h-index, an index that is widely used (and misused) in research assessments.”

- While it is acknowledged that none of the signal indicators provide definitive proof of misconduct, the manuscript would benefit from a more detailed discussion on the potential for false positives, particularly regarding legitimate scientists from less developed countries or rapidly emerging fields.

Reply: This is already clearly stated in the paper. I have re-emphasized that “Careful scrutiny is required on a case-by-case basis, to differentiate the truly stellar scientists from those who have cut corners or even engaged in fraudulent behavior to achieve such extreme metrics so fast.”

Results and discussion

-Figure 1 is informative, but its layout makes it somewhat difficult to interpret at a glance. Adding clearer visual separators, such as light grey horizontal lines between entries, could greatly improve readability. This would help readers more easily distinguish between different authors and the associated signal indicators.

Reply: As suggested, I have added grid lines between entries, and this has improved both figures 1 and 3.

- In the section stating "Self-citations and citations per citing paper were much higher in the ultra-precocious cohort than in the full list of top-cited authors and other authors were in the middle, closer if anything to the ultra-precocious cohort," it would be beneficial to also report authorship positions (e.g., first, last, single authorship) to better understand the roles and contributions associated with these citation patterns.

Reply: Thank you for suggesting this. Information on citations to papers where these scientists are single, first, or senior authors has now been added to table 2. Moreover, the proportion of citations that are made to authors where the scientist is single or first author seems to be an additional criterion that is enriched in ultra-precocious impact authors and is also frequent among those who have had retractions. The Methods have been revised to add this criterion, the Results have been revised also to include it and figures 1,2, and 3 have been edited also to include it.

-The discussion would benefit from a more nuanced exploration of alternative explanations for ultra-precocious citation patterns. Potential factors to consider include the rapid development of highly cited emerging fields, the prevalence of international hyper-authorship practices, and the effects of large-scale collaborative projects on citation accumulation.

Reply: I have added “Moreover, rapid development of highly cited emerging fields, increased prevalence of hyper-authorship practices, and large-scale collaborative projects may contribute to the emergence of ultra-precocious citation patterns.”

Other comments

While revising your submission, please upload your figure files to the Preflight Analysis and Conversion Engine (PACE) digital diagnostic tool, https://pacev2.apexcovantage.com/. PACE helps ensure that figures meet PLOS requirements. To use PACE, you must first register as a user. Registration is free. Then, login and navigate to the UPLOAD tab, where you will find detailed instructions on how to use the tool. If you encounter any issues or have any questions when using PACE, please email PLOS at figures@plos.org. Please note that Supporting Information files do not need this step.

Reply: done.

Thank you again for the very helpful feedback on the original submission. Having made these changes, I hope the manuscript will be acceptable for publication in the current version.

Sincerely

John Ioannidis
